# In Response

**DOI:** 10.4269/ajtmh.15-0777b

**Published:** 2016-02-03

**Authors:** James Nataro, O. Colin Stine

**Affiliations:** Department of Pediatrics University of Virginia Charlottesville, VA E-mail: jpn2r@virginia.edu; Department of Epidemiology and Public Health University of Maryland Baltimore, MD E-mail: ostin001@umaryland.edu

Dear Sir:

We agree with Okeke and others^1^ that undiscovered enteroinvasive *Escherichia coli* (EIEC), as well as unidentified *Shigella* infections, may both account for the increased number of cases detected by Lindsay and others.^2^ However, we emphasize that the relative proportion of the two pathogens detected depends on the *ipaH* allele that is examined. The allele used by Lindsay and others^2^ is the same allele that was used by Sahl and others^3^; this allele resides on the *Shigella* chromosome and is not present in any EIEC isolates that have been sequenced to date (D. Rasko, unpublished data). We believe, therefore, that at least the majority of the excess detected cases are likely to be caused by *Shigella* spp.
